# Oxidative Stress and Inflammation in Cancer

**DOI:** 10.3390/antiox13111403

**Published:** 2024-11-15

**Authors:** Daniela Sorriento

**Affiliations:** Department of Advanced Biomedical Sciences, Federico II University, 80131 Napoli, Italy; daniela.sorriento@unina.it

Reactive oxygen species (ROS) are important signaling molecules, physiologically synthesized by oxygen metabolism. Their role in the regulation of cell biology strictly depends on cellular levels: at low and moderate levels, ROS activate specific cellular responses and promote cell survival but at high levels they induce oxidative stress. The imbalance between the production of ROS and their elimination by antioxidant mechanisms contributes to the development of several pathological conditions, including cancer [1,2]. In cancer cells, excessive ROS production, due to increased metabolic rate and gene mutation, represents an active player in sustaining tumor development [3,4]. Indeed, ROS have been shown to affect cancer metabolism and inflammation, and to regulate cell survival [5,6,7]. Therefore, targeting ROS in cancer by acting on their generation machineries or on the redox adaptation mechanisms, could represent a potential therapeutic strategy, also to overcome drug resistance [8]. This Special issue collected several scientific contributions in this field which increase the knowledge on dysregulated molecular mechanisms in oxidative stress-dependent cancer development suggesting novel diagnostic and therapeutic strategies.

Research in the field has been rapidly progressing in the last decade, also due to the use of cutting-edge technologies. Indeed, it has been revealed the possibility of combining mechanistic modeling and omics data to decipher overall redox status in tumor tissues, allowing to gain mechanistic insights and develop effective and selective therapeutics [8] (Contribution 1). Among ROS effects in cancer, the activation of transcription factors deserves a note, since they regulate key processes such as survival, angiogenesis, inflammation, adaptive metabolism, and resistance to therapies [9,10]. In this context, NFkB exerts a key role since it regulates several processes involved in cancer development. Moreover, this transcription factor is both trigger and target of ROS contributing to worsening redox status in tumor tissues [3,11,12]. NFkB induces the release of pro-inflammatory cytokines favoring an inflammatory tumor environment which predisposes to cancer development and promotes tumorigenesis [13]. This evidence proposes NFkB as the crossroads between oxidative stress and inflammation in cancer and NFkB targeting as a promising strategy for cancer treatment, acting on both ROS production and tumor inflammation [14,15]. Today, several bioactive natural compounds [16,17,18,19,20] and small molecules [21,22,23,24] have been tested for their anti-tumor activity due to the regulation of ROS/NFkB signaling but further investigations are needed to translate these results to clinic.

Several risk factors have a huge impact on cancer development: smoking, obesity and overweight, physical inactivity, alcohol use, environmental pollution. Among them, obesity is associated with several common cancer types due to the generation of excessive ROS production and promotion of an inflammatory state [25]. Indeed, inflammatory changes in adipose tissue during obesity, activates endocrine signals which can affect cancer cell behavior, and macrophages and adipocytes in tumor microenvironment cooperate to trigger paracrine mechanisms that promote cancer development. These effects are effectively counteracted by antioxidants which can modulate both endocrine and paracrine signaling (Contribution 2).

Since oxidative stress can favor cancer development and progression, it could be considered both therapeutic target and biomarker for this condition. As biomarker, a correlation between the increase in the expression of oxidative stress markers (PARP-1, NOX1, NOX2, eNOS and iNOS) and low survival rates has been revealed in pancreatic cancer (Contribution 3). As therapeutic target, several substances of natural origin with antioxidant properties have been proposed to reduce the risk of cancer development or delay its progression (phenols, flavonoids, terpenoids, alkaloids) (Contribution 4). The beneficial effects of polyphenols from plants to modulate oxidative stress and inflammation in cancer are well established, acting as scavengers of ROS [26]. However, their pharmacological application is limited due to the scarce water solubility and bioavailability of many natural compounds. Therefore, novel drug delivery systems using biocompatible materials have been developed which would allow the use of polyphenols as therapeutics to inhibit cancer progression (Contribution 5). In addition to anti-cancer effects, these polyphenols are also able to reduce the toxicity of bisphenol A, that is used in many areas of industry to produce plastics, and has teratogenic, mutagenic, and carcinogenic effects due to the induction of ROS generation (Contribution 6). Also, piperine, a natural alkaloidal pungent product present in pepper plants, has been shown to be effective in cancer treatment due to its anti-inflammatory and anti-metastasis properties. Indeed, piperine inhibits angiogenesis of endothelial cells by activating ERK1/2 and AKT pathways which, in turn, downregulate IL-8 expression (Contribution 7). However, clinical trials with natural substances often displayed controversial results, with no benefit for human health or even adverse consequences, as occurs for carotenoids (Contribution 8). Therefore, further studies are needed for a better characterization of target patients and effective dosages.

The treatment options for cancer commonly include surgery, radiation, and chemotherapy, but the recipient cells of these treatments include healthy cells leading to the onset of several acute or chronic side effects. Indeed, many drugs are effective in delaying tumor progression but can also induce damage in many vital organs (heart, kidney, lungs, reproductive organs) impacting on patients care [27]. Therefore, understanding the mechanisms of chemotherapy-induced organ toxicity could be useful to provide effective, individualized management of cancer patient. A crosstalk between macrophages and cardiac cells has been identified that participates in cardiac damage in response to Doxorubicin treatment. Thus, oncologic patients undergoing anthracycline therapy with high cardiovascular risks could be treated with low doses of β-blockers before the expression of clinical signs of cardiotoxicity to prevent catecholamines-dependent activation of cardiac cell death [28]. Also, Cisplatin has several side effects, including gonadotoxicity and infertility, and oxidative stress has been implicated in the pathogenesis of cisplatin-induced testicular dysfunction. Kinetin, an N6-substituted adenine derivative, could ameliorate cisplatin-induced reproductive toxicity and organ damage, reducing oxidative stress, inflammation and apoptosis (Contribution 9).

The association of chemotherapy and immunotherapy (chemo-immunotherapy) is recently emerging as therapeutic option in the treatment of some tumors, including melanoma and lung cancer, since the cytotoxic effect of chemotherapy favors the action of immunotherapy. However, the metabolic reprogramming, genomic instability and oxidative stress support immunosuppression which allow tumor cell to escape attack and elimination by immune cells, as it occurs in glioblastoma (Contribution 10). Thus, further investigations on the mechanisms of immunosuppression and the interactions of cancer and immune cells are essential to improve therapies and patient’s outcomes.

Despite the growing progress in the treatment of cancer, drug resistance still represents a great challenge which can arise from genetic, epigenetic, and microenvironmental factors [29]. The underpinning mechanisms include drug inactivation, drug efflux, drug target alterations, cell death inhibition, compensatory pathways activation, DNA repair enhancement, and tumor heterogeneity [30]. Among them, ferroptosis is a novel type of regulated cell death, due to the accumulation of iron and lipid peroxides. The downregulation of ferroptosis, that occurs in cancer cells, is closely associated with drug resistance and metastasis in cancer [31]. Thus, the induction of ferroptosis should restore the sensitivity of cancer cells to treatments, as it was shown in epithelial ovarian cancer [32]. In these cells, the treatment with ferroptotic inducers can modify intercellular communication by small extracellular vesicles (sEV), inducing cell death in recipient cells (Contribution 11).

In conclusion, the scientific contributions collected in this Special Issue support the proof of concept that oxidative stress and inflammation are closely linked to pathophysiological mechanisms which lead to cancer (Figure 1). Indeed, increased ROS levels induce oxidative stress and inflammation, which, in turn, favor tumorigenesis, angiogenesis, invasion and metastasis. Thus, future studies should be focused on the discovery of novel approaches aimed at blocking both oxidative stress and tumor inflammation. As molecular therapeutic target NFkB seems to be the most promising, therefore the identification of specific, non-toxic inhibitors of this transcription factor are essential. Also, the use of natural compounds with both anti-inflammatory and antioxidant properties could be an additional therapeutic approach in long-term management of cancer.

## Figures and Tables

**Figure 1:**
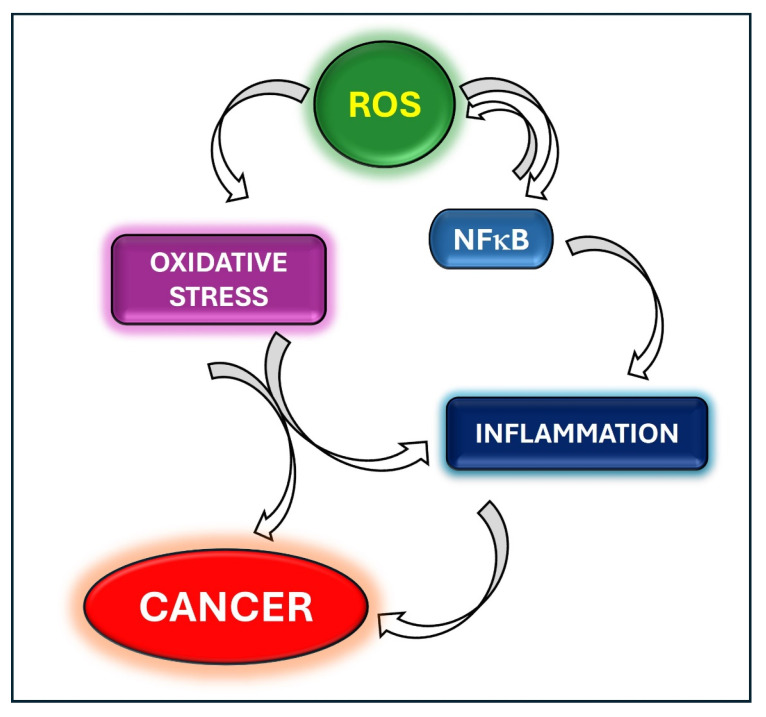
The crosstalk between inflammation and oxidative stress in cancer.
